# Hypothesis: Young infant bone strength is a multifactorial trait

**DOI:** 10.1097/MD.0000000000041701

**Published:** 2025-03-07

**Authors:** Marvin Miller

**Affiliations:** a Department of Pediatrics, Wright State University Boonshoft School of Medicine, Dayton, OH.

**Keywords:** contested child abuse cases, metabolic bone disease of infancy, multifactorial, temporary brittle bone disease, Utah Paradigm

## Abstract

Bone strength has been assumed to be relatively similar in young infants born at term. While prematurity has long been known as a risk factor for temporary bone fragility, few other factors have been appreciated that might predispose to young infant bone fragility. Moreover, young infants who present with unexplained fractures are often diagnosed as victims of child abuse based on alleged pathognomonic X-ray findings. However, review of cases of young infants with unexplained fractures often suggests child abuse is unlikely as there is often no bruising or other injuries that would be expected in these infants. The Utah Paradigm is the contemporary model of bone physiology that allows for evaluation of factors that may affect bone strength. Application of the Utah Paradigm to these cases reveals multiple, previously unappreciated, and plausible risk factors to explain the temporary bone fragility in these cases. These risk factors include decreased fetal bone loading from decreased fetal movement, maternal vitamin D deficiency, fetal exposure to drugs that can decrease bone strength, prematurity, hypermobile Ehlers Danlos Syndrome, and gestational diabetes mellitus. It is thus concluded that young infant bone strength is a multifactorial trait. Infants with unexplained fractures and bone fragility from these risk factors in which child abuse is unlikely have a recently described condition called metabolic bone disease of infancy.

**Key points**

1. Multiple factors, mostly fetal in origin, can cause young infant bone fragility.
2. The Utah Paradigm is the model of bone physiology used to evaluate these factors.
3. The hypothesis is put forth that young infant bone strength is a multifactorial trait.
4. This hypothesis has implications in the evaluation of infants with unexplained fractures.

## 1. Introduction

In 2003 I published an Editorial in the journal Bone entitled “Temporary Brittle Bone Disease: All Bones Are Not Created Equal.”^[1]^ In that Editorial the novel idea that fetal bone loading was a critical determinant of fetal and young infant bone strength was presented. It was suggested that decreased fetal bone loading was the underlying cause of temporary brittle bone disease (TBBD) and that in contested cases of child abuse the diagnosis of TBBD from decreased fetal bone loading should be considered.^[2]^ More often the decreased fetal bone loading is from intrauterine confinement, but it can also result from maternal use of drugs that cause decreased fetal movement.^[3]^ The hallmark clinical findings of TBBD are multiple unexplained fractures (MUF) in the first 6 months of life and the lack of bruising and internal injuries that would be suspected if this were child abuse and the infant had normal strength bones. The evidence that TBBD was an infant bone fragility disorder was based on the studies that showed infants with TBBD had low bone density and unfavorable bone architecture compared to control infants.^[2,4]^

Over the past 30 years I have evaluated over 500 infants with MUF in contested cases of child abuse, and since 2003 through these cases I have now appreciated additional factors that can cause decreased fetal and young infant bone strength as detailed in Table 1.

**Table 1:** Factors that can affect young infant bone strength.

Fetal/gestational factors
1. Decreased fetal bone loading from intrauterine confinement (example: twins, breech presentation, LGA fetus)
2. Decreased fetal bone loading from use of drugs by mother during pregnancy (example: methadone)
3. Prematurity
4. Maternal VDD during pregnancy
5. Inadequate provision of essential bone nutrients (vitamin D, calcium, phosphate, protein) to fetus (example: malnutrition in mother, poor maternal diet, mother who had bariatric surgery)
6. Use of drugs by mother during pregnancy that can interfere with calcium absorption (example: acid-lowering drugs like Zantac)
7. Use of drugs by mother during pregnancy that can interfere with phosphate absorption (example: calcium carbonate drugs)
8. Use of drugs by mother during pregnancy that can decrease fetal bone quality (example: magnesium, SSRIs)
9. Gestational diabetes
10. IUGR
11. h-EDS in either/both parents
12. Genetic disorders
Postnatal factors
1. Infant VDD
2. Use of drugs by infant that can interfere with calcium absorption (example: acid-lowering drugs like Zantac or Reglan)
3. FTT/infant malnutrition (example: malabsorption)
4. Infant immobilization (example: being on a ventilator)

This table lists the fetal and gestational factors that can cause young infant bone fragility in MBDI. These are the major contributing factors to MBDI and are no longer operative once the fetus is born. This table also lists several postnatal factors that can also contribute to young infant bone fragility, and these are minor contributing factors to MBDI and less frequent than the more critical fetal and gestational factors.

FTT = failure to thrive, h-EDS = hypermobile type of Ehlers Danlos Syndrome, IUGR = intrauterine growth retardation, LGA = large for gestational age, SSRIs = selective serotonin reuptake inhibitors, VDD = vitamin D deficiency.

## 2. Hypothesis

Based on the observation that there are now multiple factors that can affect young infant bone strength and predispose to fragility fractures, it is hypothesized that young infant bone strength is a multifactorial trait.

Thus, when unexplained fractures occur in a young infant, the possibility exists that these are fragility fractures from 1 or more of these factors that can affect bone strength. It has been suggested that the name for this multifactorial, infant bone fragility disorder is “metabolic bone disease of infancy” (MBDI).^[5]^

To justify that this hypothesis is plausible and that the entity MBDI exists, the following sections will be presented in this review:

- An evolution of the observations beginning in 1993 that suggested the hypothesis of the existence of a previously undefined infant bone fragility disorder that could be confused with child abuse.
- Evidence that:
  - this hypothesis has a scientific underpinning using the Utah Paradigm
  - this hypothesis explains the burst of fragility fractures that can be seen in young infancy based on the rapid bone growth of the fetus
  - there are multiple clinical observations that support this hypothesis and that child abuse is unlikely
- A description of the multiple risk factors for bone fragility and the studies that support such
- A justification for the existence of the entity MBDI based on the existence of these multiple bone fragility risk factors
- Implications of the existence of MBDI as it relates to child abuse diagnoses

### 2.1. Evolution of the hypothesis

Since the 1970s child abuse pediatricians (CAPs) have posited that infants who present with MUF in which the parent has no explanation for the fractures such as a traumatic event, are necessarily the victims of child abuse.^[6]^ More recently pediatric radiologists have popularized the idea that there are specific types of fractures like posterior rib fractures and classical metaphyseal lesions that are highly specific for child abuse.^[7,8]^ However, several studies have been published in the past 30 years which challenge the dogmas of child abuse pediatricians and pediatric radiologists.

In 1993, Paterson et al’s^[9]^ study on variant osteogenesis imperfecta (OI) that he called TBBD challenged these dogmas. Paterson’s review of 39 cases of infants with MUF in which the alleged perpetrator denied wrongdoing and the OI testing was normal revealed several risk factors associated with young infant bone fragility in contested child abuse cases of infants with MUF including prematurity, twins, and parents with joint hypermobility.

Paterson was convinced that these infants were unlikely abused as soft tissue and internal organ injury in these infants were conspicuously absent. Moreover, when these infants were returned to their parents, follow-up studies showed they did well.^[10]^

In 1999, Miller and Hangartner’s^[2]^ study established fetal bone loading as a critical determinant of young infant bone strength and explained why prematurity and twins were associated with TBBD. This was the first study that determined a true measure of bone strength, CT bone density, in both infants with MUF and controls. Miller suggested that any situation that caused decreased fetal immobilization such as intrauterine confinement or the maternal use of drugs during pregnancy that led to decreased fetal movement such as opioids could lead to weakened fetal and young infant bones and a high risk for fragility fractures.^[3]^

In 2008, Varghese et al’s^[4]^ article showed that bone architecture, yet another determinant of bone strength, was less favorable in infants with TBBD compared to controls.

In 2008, the article by Keller and Barnes^[11]^ suggested maternal vitamin D deficiency (VDD) was yet another risk factor for fetal and young infant bone fragility.

The article by Ayoub et al and the article by Miller and Mirkin provided compelling findings that many CMLs were indeed not fractures, but rather regions of non-mineralized bone, a direct challenge to the accepted thinking that CMLs were pathognomonic of child abuse.^[12,13]^

In 2017, Holick et al evaluated VDD and hypermobile Ehlers Danlos Syndrome (h-EDS) as risk factors for bone fragility in 72 contested child abuse cases of infants with MUF and found a striking overrepresentation of both, no other risk factors for bone fragility were evaluated. Infants had 1 or both risk factors. In 67 cases (93%) there was clinical evidence of h-EDS and/or a family history with a confirmed diagnosis of at least 1 parent with h-EDS. Vitamin D deficiency (VDD = 25OH-Vitamin D level < 20 ng/mL) was found in 31 of the 47 (66 %) infants tested and in 16 of the 30 (53 %) mothers tested.^[14]^

In the 72 infants in this study 5 infants had only VDD; and of the 43 infants with h-EDS who were tested, 27 had VDD.

In 2019, Miller et al^[5]^ published a case series of 75 infants with MUF in contested cases of child abuse where multiple risk factors for bone fragility were identified, all consistent with the Utah paradigm. These risk factors and their frequency included:

1. Decreased fetal bone loading (84%)
2. VDD in mother (65%)
3. VDD in infant (22%)
4. Gestational diabetes mellitus (8%)
5. Prematurity (25%)
6. h-EDS (20%)
7. Fetal drug exposures that adversely affect bone strength (63%)

In the Miller et al study, “2 infants had no risk factors, 10 had 1, 28 had 2, 23 had 3, 10 had 4, and 2 had 5.” Cases in this study were not ascertained if they had risk factors, but rather if they had abnormal X-rays showing poor bone mineralization. All 75 had abnormal X-rays, and 73 of the 75 had at least 1 risk factor.

In both of these studies the risk factors for bone fragility were greater than historical controls obtained from the literature as noted below:

1. Decreased fetal bone loading: 4% to 14%^[15]^
2. VDD in mother: Black, 29%; White, 5%^[16]^
3. VDD in infant: Black, 45%; White, 9%^[16]^
4. Gestational diabetes mellitus: 5%^[17]^
5. Prematurity: 10%^[18]^
6. h-EDS: 0.2%^[19]^
7. Fetal drug exposures that adversely affect bone strength: 37%^[20]^

The striking observation about the Miller et al study is the approach these authors used in recognizing these risk factors in contested cases of child abuse in young infants with MUF. Child abuse was deemed highly unlikely in these cases based on the lack of other injuries such as bruising and internal organ injury, even though the CAP reflexively diagnosed child abuse when the parents could not offer an explanation for the fractures.

The authors then evaluated the pregnancy history, family history, medical history, and imaging studies of the young infants. From this comprehensive review it was apparent that certain findings were overrepresented in these contested cases which proved to have validity as risk factors for bone fragility as there was a scientific basis for them based on the Utah Paradigm and the existing medical literature. Moreover, the radiographic findings in all these cases showed poor bone mineralization consistent with healing rickets, thus confirming that these young infants had a bone fragility disorder.

The clinical findings of both TBBD and MBDI are the same. While my original clinical study on TBBD suggested it was only decreased fetal bone loading as the cause of the fragility fractures in these infants with MUF, MBDI encompasses all of these more recently appreciated risk factors, and is thus a more comprehensive and current term.

The Utah Paradigm was critical to my understanding these observations and will be described in the following section.^[21]^

### 2.2. Evidence for the hypothesis

There are multiple observations that support the above hypothesis including the following:

- The Utah Paradigm explains the risk factors

**Figure 1.**
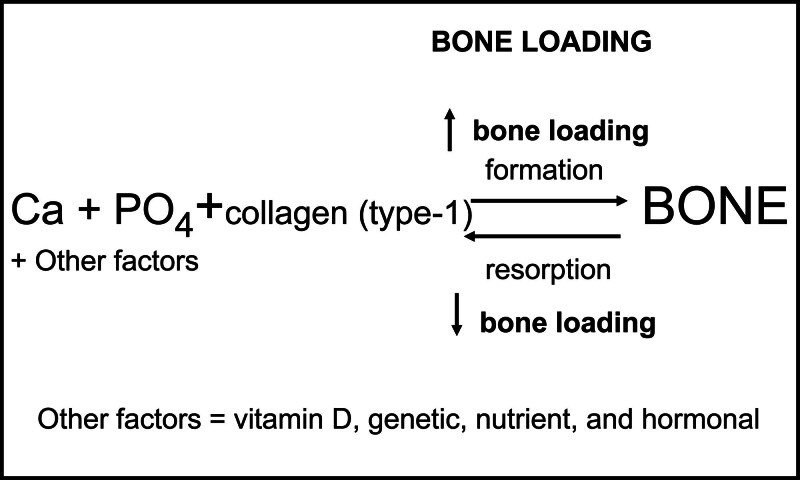
The Utah Paradigm is the contemporary model of bone physiology that can be used to predict factors that can affect bone strength. While the essential nutrients (calcium, phosphate, vitamin D, and protein) must be provided for bone formation, the catalyst to produce and strengthen bone is the load the bone experiences in its environment.

The Utah Paradigm is the contemporary model of bone physiology popularized by visionary bone scientist Dr Harold Frost that can be used to understand factors that determine bone strength and weakness at any age, as shown in Figure 1.^[2,3,21]^

**Figure 2.**
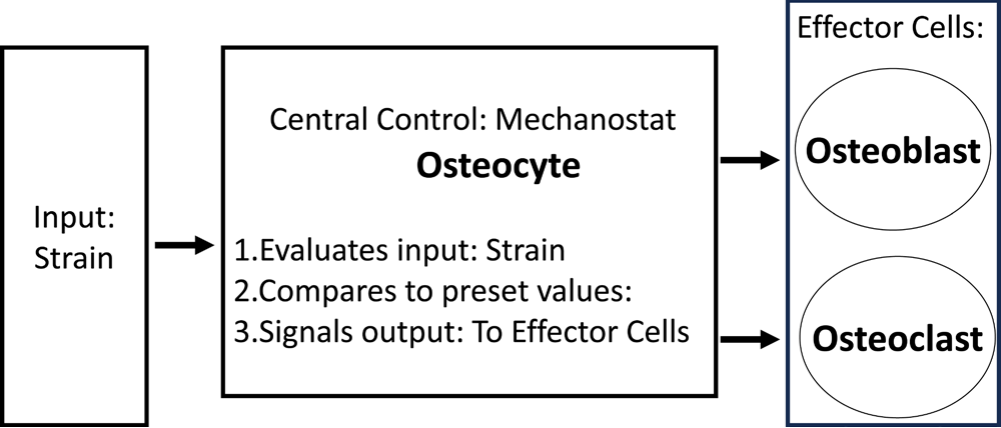
A stable bone strength is determined by the input of strain* on the bone. Strain is evaluated by the mechanostat, the osteocyte. If the strain remains constant, the bone strength is unchanged. If, however, there is a decrease or increase in strain, the osteocyte will register this and send signals to the effector cells (osteoblasts and osteoclasts) to change the bone strength in accord with the newly appreciated strain. A decrease in strain will cause a decrease in bone strength. An increase in strain will cause an increase in bone strength. Bone strength can be increased or decreased by the effector cells through changes in bone density and/or bone architecture. *Strain = change in length of a long bone/length of the long bone.

This model recognizes there are essential nutrients that produce bone including calcium, phosphate, vitamin D, and protein, but the centerpiece of the Utah Paradigm is the concept that bone loading is the critical determinant of bone strength. The Utah Paradigm posits a regulatory system within bone that produces a bone strength that is appropriate for the load placed on the bone that is accomplished through a coordination of activities between the 3 types of bone cells: osteocytes, osteoblasts, and osteoclasts as shown in Figure 2.^[21]^

Osteocytes are the mechanosensory cells that detect the load the bone experiences and are thus the mechanostat of the bone.^[21]^ The osteocyte is buried in lacunae within bone and has multiple, thin cellular projections bathed in fluid that can detect even the slightest change in strain produced by a force applied to the bone. Strain is the proportional change in length (change in length/length) caused by a force that can be from compression, tension, or shearing loads. Because bone is composed of both a brittle material (mineral) and an elastic 1 (type 1 collagen), it has the ability to bend, but not break under ordinary circumstances, even when the slightest force is applied to them to produce a strain.

As shown in Figure 2, the osteocyte is able to discern any change in strain, and then signal the effector cells, osteoblasts and osteoclasts, to change the bone strength so that it is now aligned with the new strain. These changes in bone strength can occur by changes in bone density and or bone architecture.

**Figure 3.**
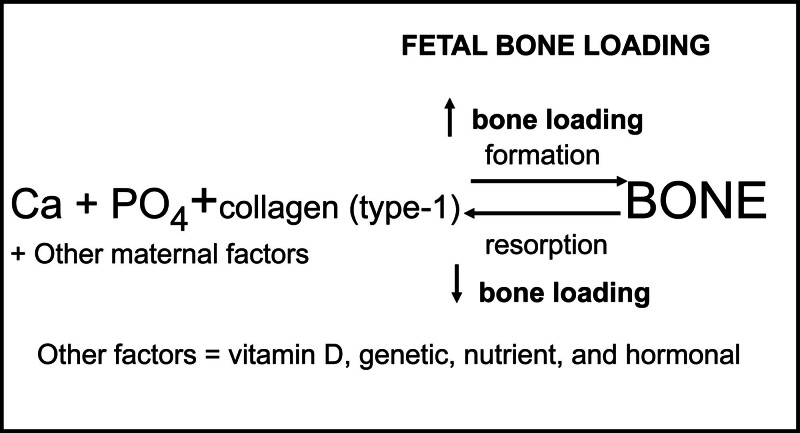
The Utah Paradigm can be applied to the feta l time period and thus can used to predict factors that can affect fetal bone strength. During pregnancy the mother needs to provide essential bone nutrients (calcium, phosphate, vitamin D, and protein) to the fetus. However, the major catalyst to produce and strengthen bone during the fetal time period is the bone loading that the fetal skeleton experiences through fetal movements that strike the uterine wall.

I have maintained that the Utah Paradigm also applies to the fetus as shown in Figure 3. This system for regulating fetal bone strength is established and fully functional during the second and third trimesters of pregnancy.^[2,3,5]^ Just as bone loading is critical in maintaining bone strength in postnatal life, bone loading is likewise critical in determining bone strength in the fetus and this is accomplished mainly through fetal movement.

B. The unique character of fetal and young infant bone strength

The fetus and young infant have a unique set of bone strength determinants that are different from adults and older children. Young infant bone strength is, in great part, determined by the bone strength of the fetus at the time of delivery, and it is the fetal bone strength at the time of delivery that has unique bone strength determinants. This unusual situation is from the intricate relationship of the fetus with the gravid mother. First, the mother’s duration of the pregnancy, her diet, her health, and the medications she takes during pregnancy can affect the strength of the fetal skeleton. Second, the intrauterine environment of the fetus normally offers ample opportunity for movement and kicking against the uterus, and this bone loading is the catalyst for promoting the development of normal fetal bone strength. Decreased fetal movement leads to decreased fetal bone loading and thus decreased fetal bone strength.

Most importantly, the magnitude of effect of any adverse environmental factor that affects bone strength is directly related to the rate of growth of the skeleton at that particular time. The most rapid period of human bone growth is in the third trimester of pregnancy when fetal linear bone growth is about 100 cm per year, and the next most rapid bone growth occurs in the neonatal time period when linear bone growth is about 50 cm per year.^[22]^

It is during times of most rapid bone growth that the human skeleton is at greatest risk to become undermineralized if one of the critical factors that determines mineralization is compromised. As previously noted, the Utah Paradigm posits that bone loading and calcium, phosphate, vitamin D, and protein availability determine bone strength. Thus, during the most rapid period of skeletal growth, the third trimester of the human pregnancy, decreased fetal bone loading and insufficient maternal provision of calcium, phosphate, vitamin D, and protein to the fetus can result in a weaker skeleton in the fetus, and therefore the young infant.

C. Clinical observations that align with the hypothesis

There are multiple clinical observations about TBBD, and thus also MBDI, that support this hypothesis:

- The incredibly high number of fractures without significant bruising or internal organ injury which would be expected if bone strength were normal.

In 1 series of 65 young infants with TBBD, the mean number of fractures was 11 (range = 3–26 fractures), and the mean age of presentation was 9 weeks (range = 2–24 weeks).^[3]^ In 1 series of 75 infants with MBDI, the mean number of fractures was 10 (range = 1–36 fractures), and 36% of the infants had 10 or more fractures. The mean age of presentation was 10 weeks (range = 1–36 weeks), and 82% of the infants presented at or before 16 weeks.^[5]^

The lack of bruising or other internal injury observations is compelling evidence that the fractures in TBBD/MBDI are fragility fractures.^[2,5,9]^ Several studies report a strong association between soft tissue injury including bruising and fractures in infants injured by intentional injury.^[23,24]^

ii The high number of cases with 4 or more rib fractures and lack of internal thoracic injury

Multiple rib fractures in ribs of normal strength are strongly associated with a high likelihood of finding severe internal lung injury (lung contusion, pneumothorax, and hemothorax) as the forces needed to cause multiple rib fractures is extremely high. Garcia found that whenever there were 4 or more rib fractures in infants with normal strength ribs, there was always severe internal thoracic injury and severe respiratory distress.^[25]^ In the Garcia study there were 14 deaths among the 33 infants with rib fractures, and 7 of the 33 infants with rib fractures were from child abuse.

The lack of such internal thoracic injury and lack of respiratory distress in the over 200 cases I have evaluated with MUF and 4 or more rib fractures suggests the forces that caused the rib fractures were trivial. Moreover, when the healing rib fractures were dated using the Sanchez method, many date back to birth and are consistent with birth injury fractures.^[26,27]^

iii The occurrence of fractures in infants with TBBD in the hospital

Paterson described 5 infants with TBBD where the fractures occurred while in the hospital, suggesting the fractures were fragility fractures.^[28]^

iv Follow-up of infants with TBBD returned to their parents shows normal development

The long-term follow-up (mean of 6.9 years, range: 1–17 years) of 61 infants with TBBD in contested cases of child abuse in which Paterson’s testimony in legal proceedings led to their return to parents showed all these children thrived without any evidence of subsequent injury.^[10]^

v The narrow window of presentation in the first 6 months of life with few fractures or cases after 6 months of age

The presentation of the multiple fragility fractures in the first 6 months of life with few after 6 months of age suggests that the factors that cause MBDI are fetal in origin.^[3,5]^ That these risk factors are fetal in origin explains the above observations, especially the high growth rate of bones in the fetal time period and the increased susceptibility for bone fragility.

## 3. The risk factors for young infant bone fragility

The critical and more common risk factors for young infant bone fragility are fetal or gestational in origin. There are several postnatal factors that could also affect young infant bone strength.

Table 1 lists the fetal and gestational factors that can cause young infant bone fragility in MBDI. These factors are no longer operative once the fetus is delivered, and the bone fragility typically is present for the first 6 months of life.

Table 1 also lists several postnatal factors that can also contribute to young infant bone fragility, especially if they occur shortly after birth, and include vitamin D deficiency, failure to thrive, being on drugs that can unfavorably affect bone strength, or immobilization from being on a respirator. Postnatal factors are minor factors in MBDI, and less frequent than the more critical fetal and gestational factors.

### 3.1. Fetal and gestational factors

#### 3.1.1. Decreased fetal bone loading

The catalyst for increasing fetal bone strength is fetal bone loading that results from the fetal movement and especially from kicking the mother’s uterine wall.^[2,3]^ Multiple human studies have demonstrated this in various settings of intrauterine confinement including the twin pregnancy, the breech presentation and the infant who is large for gestational age (LGA).^[29–32]^ Human and experimental animal studies have shown that fetuses and pups who had decreased fetal movement as evidenced by a short umbilical cord have decreased bone strength.^[33–35]^

#### 3.1.2. Prematurity

Gestational age is an important determinant of young infant bone strength as prematurity can lead to the bone disease of prematurity (BDP) and an increased risk for fragility fractures.^[36–39]^ The etiology of BDP is likely several fold, resulting from insufficient dietary calcium and phosphate, decreased bone loading, and exposure to hypercalciuric drugs like furosemide and theophylline. The intrauterine environment provides far greater bone loading from fetal kicking of the uterus and free movement compared to the extrauterine environment of a relative immobilization while lying in a NICU crib.^[40]^

#### 3.1.3. Maternal vitamin D deficiency (VDD)

In 2008, Keller and Barnes^[11]^ published a series of young infants with fractures and bone fragility that they attributed to VDD in the mother during the pregnancy. In this study they presented radiographs showing healing rickets indicating the fractures in these infants were fragility fractures. VDD in a pregnant mother can have 2 effects that could decrease fetal bone strength. First, the mother’s absorption of calcium from her GI tract would be less than if she were VD sufficient, and thus the fetus would be exposed to less calcium which could lead to poor fetal bone mineralization. Second, the fetus would be VDD and this would compromise osteoblast function as vitamin D needs to be bound to the vitamin D receptor of osteoblasts for the osteoblast to normally mineralize the fetal bone.

Subsequent cases series of young infants with fractures and VDD in the infant or mother have been reported.^[5,14]^ Cases of congenital rickets from maternal VDD have been reported.^[41]^

#### 3.1.4. Other maternal dietary deficiencies during pregnancy

In addition to VDD, a maternal dietary deficiency of protein, calcium, or phosphate could lead to poor fetal mineralization. Raman et al^[42]^ showed that malnourished mothers who were given calcium supplements during pregnancy had newborns with greater bone density. Pregnant women who have had bariatric surgery are at risk for dietary deficiencies of the 4 essential nutrients required for developing normal bone strength: protein, VD, calcium and phosphate. Infants with fragility fractures and healing rickets have been described in the offspring of mothers who have had bariatric surgery.^[43]^

#### 3.1.5. Maternal use of drugs that can affect fetal bone

There are several drugs that a mother can take during pregnancy that can unfavorably affect fetal bone strength through various mechanisms and include the following:

1. Acid-lowering drugs
  Acid lowering drugs that are prescribed for heartburn during pregnancy decrease the absorption of calcium in the mother’s GI tract and therefore can decrease the amount of calcium presented to the fetus.^[44,45]^
2. Calcium-containing drugs
  Calcium containing drugs (calcium carbonate or Tums) for treatment of pregnancy heartburn can diminish the availability of phosphate to the fetus as the calcium in these drugs can combine with the phosphate in the diet, and then the calcium phosphate is lost in the stool.^[46]^
3. Magnesium (Mg)
  Mg is prescribed to mothers in pregnancy for treatment of migraines, pre-eclampsia, and hypertension for treatment of migraines or preeclampsia. Multiple studies have shown that fetal Mg exposure during pregnancy causes abnormal bone mineralization in the young infant on radiographs and an increased risk to fracture in the postnatal period.^[47–55]^ The FDA has recommended not using Mg for pre-term labor because of the adverse effects on infant bone.^[56]^
4. Selective serotonin reuptake inhibitors (SSRIs)
  SSRIs are commonly prescribed during pregnancy for treatment of anxiety and depression, and they have been shown to cause diminished bone density with increased fracture risk in human adults.^[57–59]^ SSRIs not only affect the serotonin reuptake receptors in the brain, but also the 5-HT class receptors that are critical in the differentiation and proliferation of the bone-forming osteoblasts. A serotonin receptor 5-HT2A antagonist in mice causes reduced bone mass, due to inhibition of osteoblast differentiation.^[60]^

#### 3.1.6. Gestational diabetes

Infants of diabetic mothers (IDM) have been shown to have lower bone content than controls, increased bone resorption compared to controls and lower tibial speed of sound compared to controls.^[61–63]^ Mothers with gestational diabetes have been shown to have lower 25OH-VD levels than controls.^[64]^ This relative bone weakness in IDM may be related to enhanced glycosylation of bone- containing substances, a decrease in fetal movement related to the IDM being large, or maternal VDD.^[65]^

#### 3.1.7. Intrauterine growth retardation (IUGR)

IUGR is often the result of placental insufficiency which can result in the inadequate provision of the essential nutrients for the development of normal fetal bone strength.^[66]^

#### 3.1.8. Parental and/or infant hypermobile Ehlers Danlos Syndrome (h-EDS)

Young infants born to mothers or fathers with h-EDS or joint hypermobility are at increased risk to have fragility fractures, and several reports have described this association.^[5,9,14]^ There are 2 possible explanations for this association. The first is that here is an intrinsic structural abnormality in bone.^[67]^ Even though h-EDS is an autosomal dominant connective tissue disorder, h-EDS is the 1 type of EDS for which a gene has yet to be found. The second explanation, and the 1 that is more likely, is a biomechanical explanation. As a result of the joint hypermobility in the fetus who inherits h-EDS there is decreased fetal bone loading, especially if the mother had h-EDS and had a relatively softer uterus.^[68–70]^

#### 3.1.9. Genetic disorders

Single gene disorders such as osteogenesis imperfecta, hypophosphatasia and other rare bone fragility disorders can present in young infancy and be confused for child abuse.^[71]^ Bone fragility molecular panels can be obtained to evaluate for these conditions.

### 3.2. Postnatal factors

Postnatal factors are usually minor factors that can be associated with young infant bone fragility and include the following:

#### 3.2.1. Infant VDD

Infants are prone to VDD in the immediate postnatal period especially if they are breast fed without VD supplementation.^[72]^

#### 3.2.2. Infant use of acid-lowering drugs that can interfere with calcium absorption

Infants with gastrointestinal reflux are often prescribed acid-lowering drugs like Zantac or Reglan which can cause decreased infant absorption of calcium leading to bone fragility. An increase in fracture susceptibility has been described in infants prescribed acid lowering drugs.^[73]^

#### 3.2.3. Failure to Thrive (FTT)/infant malnutrition

Dietary deficiencies in the immediate postnatal period leading to FTT for whatever reason can lead to insufficient nutrients for the development of bone strength during young infancy and can lead to lower bone strength.^[74]^

#### 3.2.4. Infant immobilization

Prolonged infant immobilization, such as being on a ventilator, can lead to disuse osteopenia.^[75]^

## 4. MBDI: A name that emphasizes the multiple risk factors for young infant bone fragility

Names of medical diagnoses evolve as additional information about these disorders becomes known. Many of the risk factors for young infant bone fragility noted in Table 1 have only been recently described in the past 25 years. I have now come to appreciate that young infant bone strength is a multifactorial characteristic akin to adult cardiovascular health where there are also multiple risk factors that determine health outcomes. Young infant bone strength has multiple determinants, both genetic and environmental, and the compromise or deficiency of 1 or more of these factors can lead to fragility fractures caused by minimal trauma that might not otherwise cause a fracture in a normal strength bone. Thus, a name is needed to describe this previously unappreciated multifactorial disorder, and I have proposed the name “Metabolic Bone Disease of Infancy.”^[5]^

## 5. Implications of the hypothesis

The idea that young infant bone strength is a multifactorial characteristic has significant implications in child abuse evaluations of infants who present with MUF in which parents and caregivers deny wrongdoing.

The approach that CAPs use in evaluating infants with MUF relies on X-ray findings to diagnose child abuse as it has been mistakenly believed that there are specific fractures that are pathognomonic for child abuse such as CMLs and posterior rib fractures. Another fallacy is that if bones show “normal mineralization” it can be inferred that there is normal bone strength. X-rays cannot determine if an infant’s bones are of normal bone strength. Thus, the reliance on X-rays for establishing a diagnosis of child abuse can lead to incorrect diagnoses. Moreover, CAPS do not use a bone physiology model such as the Utah Paradigm in their evaluation of infants with MUF.

My approach recognizes that there are no specific fracture types that are highly indicative of child abuse.^[5,12,13]^ I have applied the Utah paradigm and relevant medical literature in evaluating risk factors of bone fragility in young infants.

The difference in these 2 approaches is dramatic, as I often find an explanation for the fractures in an infant with MUF that is other than child abuse. The finding of a medical or accidental cause of fractures in an infant with MUF has far-reaching implications for the family. The incorrect diagnosis of child abuse in an infant with MUF often causes wrongful parental termination of rights and sometimes even wrongful incarceration of a parent or caregiver.

The differential diagnosis of the young infant who presents with MUF includes inflicted trauma from child abuse, accidental trauma such as from a fall, rare genetic disorders such as OI, Menkes disease and hypophosphatasia, and MBDI. My experience over the past 30 years indicates MBDI is the most common cause of MUF, and the finding of healing rickets on the skeletal survey and the appreciation of risk factors for young infant bone fragility as noted in the Table 1 would strongly suggest that diagnosis.

There are several monogenic bone disorders associated with bone fragility that can present with fractures shortly after birth. The most common genetic bone disorder that can present with MUF and occasionally be confused for child abuse is osteogenesis imperfecta (OI). Menkes disease and hypophosphatasia are other rarer genetic disorders that can present with fragility fractures in the young infant. In infants with MUF a comprehensive panel of genes can now be analyzed to exclude these relatively uncommon disorders that can present with MUF. DNA companies offer OI and bone fragility gene panels to evaluate for the rare occasion of a genetic bone fragility disorder

## 6. Conclusion

Infants with MUF whose fractures are fragility fractures often have 1 or more of the previously noted risk factors as the basis of the bone fragility. In contested cases of child abuse in infants who present with MUF child abuse physicians often assume that the infant necessarily has normal bone strength as the relevant risk factors detailed herein are not appreciated or overlooked. MBDI is suggested as a new name for this multifactorial condition. Recognizing that MBDI shows radiographic features of healing rickets, has several of the risk factors previously noted, and sometimes has abnormal laboratory findings related to bone physiology will hopefully lead to more accurate diagnoses in infants who present with MUF. This list of risk factors may be current in 2024, but it is likely that new risk factors for young infant bone fragility will be recognized when the next, future commentary on this topic appears.

## Acknowledgments

The author is grateful to Shelley Miller for her critical review of the manuscript.

## Author contributions

**Conceptualization:** Marvin Miller.

**Writing – original draft:** Marvin Miller.

**Writing – review & editing:** Marvin Miller.
